# Gall Bladder Disease and the Risk of Small Bowel Cancer—Results from a Nationwide Swedish Cohort Study

**DOI:** 10.3390/cancers14030469

**Published:** 2022-01-18

**Authors:** Louise Emilsson, Cecilia Radkiewicz, Carol E. Semrad, Amit D. Joshi, Jonas F. Ludvigsson

**Affiliations:** 1 Department of General Practice, Institute of Health and Society, University of Oslo, 0315 Oslo, Norway; 2 Department of Medical Epidemiology and Biostatistics, Karolinska Institutet, 171 77 Stockholm, Sweden; cecilia.radkiewicz@ki.se (C.R.); jonasludvigsson@yahoo.com (J.F.L.); 3 Faculty of Medicine and Health, Örebro University, 701 81 Orebro, Sweden; 4 Vårdcentralen Värmlands Nysäter and Centre for Clinical Research, County Council of Värmland, Varmlands Nysater, 661 95 Karlstad, Sweden; 5 Section of Gastroenterology Hepatology and Nutrition, Department of Medicine, University of Chicago, Chicago, IL 60637, USA; csemrad@medicine.bsd.uchicago.edu; 6 Massachusetts General Hospital-Clinical & Translational Epidemiology Unit, Harvard Medical School, Boston, MA 02115, USA; adjoshi@mgh.harvard.edu; 7 Department of Paediatrics, Örebro University Hospital, 70 185 Orebro, Sweden; 8 Department of Medicine, Columbia University College of Physicians and Surgeons, New York, NY 10032, USA

**Keywords:** intestine, neoplasm, etiology, gallbladder disease

## Abstract

**Simple Summary:**

Gallbladder disease (GBD) has been linked to small bowel cancer, but earlier reports have been based on a few cancer cases and no previous studies have adjusted for potential confounders. We report a 1.4-fold increased risk of small bowel adenocarcinoma, 1.8-fold increased risk of adenomas, and a 2.1-fold increased risk of carcinoids in patients with GBD compared to matched comparators. Absolute risks were low, however (<1 per 1000 patients followed for 10 years) for all three outcomes. We lacked detailed data on body mass index, cigarette smoking, and GBD biomarkers. Further results from an observational study, like ours, cannot establish a causal relationship or rule out the presence of residual confounding. The increased risk of carcinoid, seen 11–16 years after GBD diagnosis, may represent an etiologically plausible causal link to carcinoid development and warrants further study. The reported low absolute risks argue against targeted surveillance for small bowel cancer after GBD diagnosis. In conclusion, this study reports a moderately increased relative risk of small bowel cancers and adenomas in patients with GBD; absolute risks were however low.

**Abstract:**

Background and aims: Small bowel cancer is a rare but rising malignancy. The etiology is poorly understood and there is a need for large-scale studies. Gallbladder disease (GBD), inducing localized inflammation, has been suggested to increase small bowel cancer risk. Methods: We retrieved nationwide data from Sweden’s 28 pathology departments on all adults (age 20–79) with pathology-confirmed GBD diagnosed in 1965–2017. In total 156,390 GBD patients were matched with up to 5 matched comparators from the general population and follow-up started one year after GBD diagnosis. We used stratified Cox regression to calculate hazard ratios (HRs) for small bowel adenocarcinoma, adenomas, and carcinoids. Results: During a median follow-up of 12 years, we identified 92 small bowel adenocarcinomas, 132 adenomas, and 81 carcinoid tumors in the GBD cohort. Corresponding incidence rates were 4.8, 6.9, and 4.2 per 100,000 person-years (PY), compared to 3.2, 3.2, and 1.8 in matched comparators. The adjusted HR was 1.42 (95% CI = 1.08–1.87) for small bowel adenocarcinoma, 1.79 (95% CI = 1.41–2.27) for adenoma, and 2.07 (95% CI = 1.52–2.81) for carcinoid. The excess cancer risk was most pronounced during the first year of follow-up for adenocarcinomas and during the first six years for adenomas while for carcinoids the HR peaked 10–15 years after start of follow-up. Conclusions: In this nationwide cohort study, GBD was associated with an increased risk of small bowel cancer. The excess risk of small bowel adenocarcinoma was mainly seen during the first years of follow-up while small bowel carcinoid risk peaked 11–16 years after GBD diagnosis.

## 1. Introduction

Adenocarcinomas are malignant epithelial tumors with a glandular differentiation constituting the most common cancer subtype in the colon, rectum, small intestine, pancreas, lung, breast, prostate, and stomach. Adenocarcinomas are believed to constitute a malignant transformation of an adenoma. Carcinoids, on the other hand, arise from argentaffin cells [1]. Carcinoid incidence has been increasing [2] but survival has remained constant over the last 20 years. To our knowledge, the only report on predisposing factors for intestinal carcinoids was based on 99 cases [3]; hence, the knowledge gap is indisputable.

Given that the small bowel makes up 75% of the length of the gastrointestinal (GI) tract and 90% of the mucosal surface area, tumors in this location are surprisingly rare and only account for 2% GI malignancies [4]. Known risk factors for small bowel adenocarcinomas include sex, age, ethnicity, smoking, alcohol use, Crohn’s disease [5], and celiac disease [6]. Previous studies have shown the duodenum to be the most common location of small bowel adenocarcinomas. One possible explanation for the excess cancer rates in the duodenum is the location of the ampulla of Vater and higher concentrations of bile and its metabolites inducing duodenal inflammation [4,7,8]. Acute cholecystitis is most commonly caused by obstructive gallstones leading to inflammation in the gallbladder. Risk factors are old age, female sex, high body mass index (BMI), family history, pregnancy, diet, low physical activity, recent rapid weight loss, oral contraceptives, post-menopausal hormone use, and metabolic syndrome [9]. Two older cohort studies explored the association before year 2000 [10,11], and furthermore, some more recent case-controls studies have reported an association between GBD and small bowel cancers [12,13]; however, all these studies lacked adjustment for confounders as well as assessment of absolute risk.

In this study we aimed to investigate whether symptomatic gallbladder disease (GBD) is associated with an increased risk of developing small bowel adenocarcinoma, adenoma, and/or carcinoid tumors, also when confounders are adjusted for as well, so as to report absolute risk and incidence rates.

## 2. Methods

### 2.1. Study Population

Data from Swedish national healthcare registries were linked using the personal identity number assigned to all Swedish residents [14]. Participants with GBD were identified from the Epidemiology Strengthened by histoPathology Reports in Sweden (ESPRESSO) study representing GI biopsies from all 28 Swedish pathology departments in 1965–2017 [15]. In total, 197,548 unique individuals with non-malignant gallbladder-disease-related histopathology reports were identified in ESPRESSO and linked to the Swedish Patient Register [16], whereof 171,254 were confirmed to have undergone cholecystectomy and/or were diagnosed with GBD (i.e., any gall stone and/or cholecystitis diagnosis) within ±90 days of the histopathology report (relevant codes in Appendix A). We further excluded participants aged <20 years or ≥80 years (*n* = ?) or with a history of cancer in the liver, pancreas, biliary tract, and/or small bowel (also adenoma when it is the relevant outcome). 

### 2.2. Outcome Measure

We identified small bowel adenocarcinomas, adenomas, and carcinoids using the corresponding SnoMed codes from pathology reports (see Appendix A) [15].

### 2.3. Matched Comparators

For each GBD patient, the government agency *Statistics Sweden* randomly identified up to five comparators from the Swedish Total Population Register [17] matched by age, sex, county, and calendar year of histopathologic GBD diagnosis. Matched comparators with a history of cholecystectomy were excluded from the analysis. 

### 2.4. Follow-Up

Follow-up started 1 year (365 days) after GBD diagnosis, or corresponding date for the matched comparators, to avoid ascertainment bias, i.e., asymptomatic GBD detected during the clinical workup of small bowel cancer, or small bowel tumors causing biliary obstruction. Follow-up ended at first occurrence of either date of death, emigration, outcome (small bowel adenocarcinoma, adenoma, or carcinoid in separate analyses as defined by ESPRESSO), or administrative end of follow-up (31 December 2017).

### 2.5. Statistics

We calculated hazard ratios (HRs) using stratified Cox regression. In the stratified regression, each case is compared to his/her matched comparators and a pooled summary HR is calculated from all strata. All analyses were adjusted for categorical educational level (≤9, 10–12, ≥13, missing) [18] and comorbidity; chronic obstructive pulmonary disease (as a proxy for smoking), alcohol-related disorders [19], type 2 diabetes, obesity, and ischemic heart disease, retrieved from the patient registry (see ICD codes in Appendix A). Baseline characteristics: age, sex, educational level, and calendar year are presented as numbers and proportions. We present adjusted HRs stratified by follow-up (0- < 1 (i.e., year 1–2 after GBD diagnosis), 1- < 5, 5- < 10, 10- < 15, 15- < 20, and ≥20 years), and restricted to the 1- < 6 years after GBD date to allow for comparisons of calendar periods. Incidence rates were calculated as the number of small bowel cancer events per 100,000 person-years of follow-up. The proportional hazards assumption was verified using interaction terms with log (time). All Analyses Were Performed Using SAS 9.4.

Ethics. The current study was approved by the Stockholm Ethics Review Board 2014/1287-31/4) on 27 August 2014. The ethics review board did not require informed consent as it is a strictly register-based study [20].

## 3. Results

In total, we identified 156,390 individuals diagnosed with GBD and with at least one year of follow-up. GBD was associated with female sex (63%), COPD (8.5% vs. 0.5%), alcohol-related disorders (3.8% vs. 0.3%), and obesity (3.0% vs. 0.7%) (Table 1). The median follow-up was 12 years, ranging from 0 to 46 years.

### 3.1. Small Bowel Adenocarcinoma

**Figure 1:**
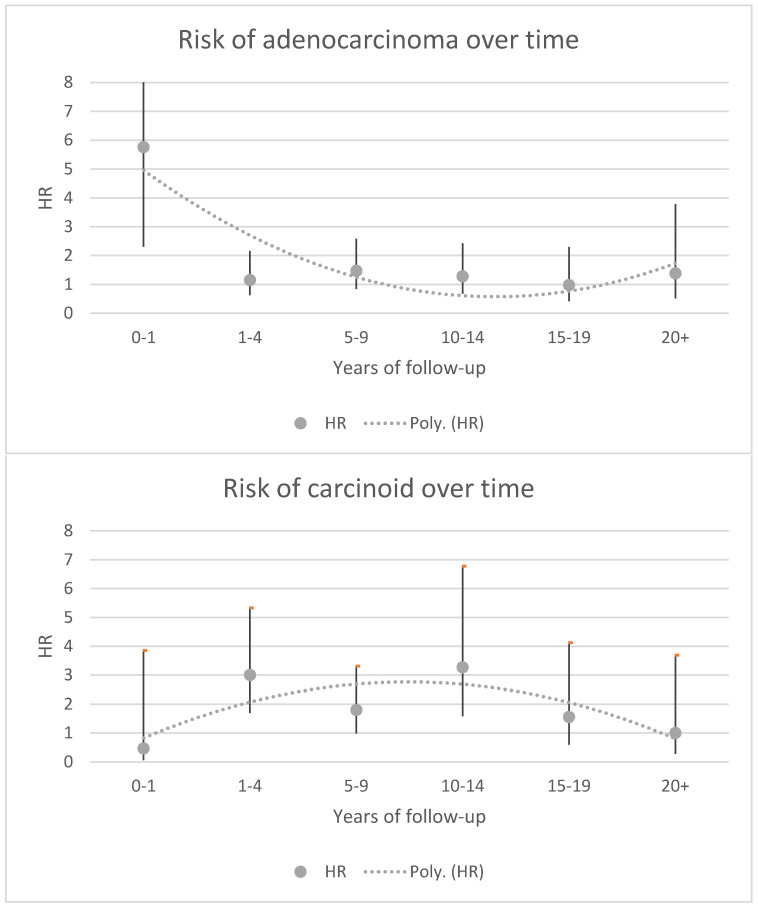
Relative risk of adenocarcinoma and carcinoid over time after study entry (i.e., time 0 equals 1 year after first gall bladder disease date). The fitted trend line represents a polynomial of 2 order.

Overall, we identified 92 small bowel adenocarcinomas in GBD patients (of those, 13 were smokers, 7 had high alcohol consumption, and 6 individuals had type 2 diabetes) compared to 260 among comparators. The corresponding HR for small bowel adenocarcinoma was 1.42 (95% CI = 1.08–1.87), with a similar HR for *duodenal* adenocarcinoma (HR = 1.43; 95% CI = 1.00–2.05). The association was particularly strong within the first year of follow-up (i.e., year 1–2 after GBD, Figure 1) and in stratified analysis it was only significant in men and individuals aged 20–39 at GBD diagnosis. The overall incidence rate was 4.8 per 100,000 PY in GBD patients vs. 3.2 in matched comparators, i.e., the absolute risk difference was 1.6 cases per 100,000 PY. The incidence rate was higher in men (7.0) than in women (3.7) with GBD (Table 2).

### 3.2. Small Bowel Adenomas

Small bowel adenomas were identified in 132 GBD patients and 260 matched comparators. The overall risk of small bowel adenoma was increased (HR = 1.79, 95% CI = 1.41–2.27). In contrast to stratified analyses for adenocarcinomas, the risk of adenomas was significantly increased in both men and women, mostly within year 1–5 after follow-up (2–6 after GBD), in older age groups (significant for all except age 20–39 years), and in individuals with less than 12 year of education (Table 3). The overall incidence rate was 6.9 in individuals with GBD vs. 3.2 in matched comparators and hence the risk difference is 3.7 additional cases per 100,000 PY.

### 3.3. Small Bowel Carcinoids–Neuro Endocrine Tumors/Carcinomas

In total, we identified 81 cases and 148 matched comparators diagnosed with carcinoids during follow-up, corresponding to an HR of 2.07 (95% CI = 1.52–2.81) and an incidence rate of 4.2 vs. 1.8 per 100,000 PY (Table 4). Among the 81 individuals diagnosed with GBD and carcinoids, 13 were smokers/had COPD, 3 had registered high alcohol consumption, 4 had type 2 diabetes, and 3 had ischemic heart disease (Table 1). The risk was similar in both men and women and showed the largest HRs during 1–5 and 10–15 years after start of follow-up (Figure 1). The HR was higher the younger the age group at GBD, and was significant in age groups 40–70; under 40 showed a strong HR that lacked statistical significance whereas there were no strong associations after age 70 (Table 4). The overall incidence rate was 4.2 in individuals with GBD vs. 1.8 in matched comparators, hence absolute risk difference is 2.4 additional cases per 100,000 PY.

## 4. Discussion

In this nationwide cohort study of more than 150,000 patients with GBD, we found a 1.4-fold increased risk of small bowel adenocarcinoma, 1.8-fold risk for small bowel adenomas, and a 2.1-fold risk of carcinoids. While excess risks for small bowel adenocarcinoma and adenomas were mainly seen during the first years of follow-up, the HRs for carcinoids peaked in a bimodal pattern at 1–4 and 10–15 years after study entry (Figure 1). An increased risk 11–16 years after diagnosis is consistent with previous knowledge of carcinogen induction time [21] and suggests a potential causal mechanism for GBD and carcinoid development; however, it should be noted that this finding is based on a total of 18 cases that developed carcinoids within this timeframe. The increased risk of adenocarcinomas seen mainly during the first year most likely represents shared risk factors and/or ascertainment bias, even though we attempted to minimize the impact from ascertainment bias by starting follow-up one year after GBD diagnosis. Potential explanations for the associated risk of small bowel cancers include microbiota imbalance. Microbiota imbalance has been hypothesized to promote colorectal carcinogenesis and some bacterial species are considered more pro-carcinogenic than others [22]. Hence, it is possible that specific microbiota that increase the risk of gallbladder infection and/or stones also affect the cancer risk in the small bowel. Another potential explanation could be that bile leakage into the small bowel may be a chemical trigger of carcinoid development. However, absolute risks were low for all outcomes, less than 10 per 100,000 PY for all studied outcomes, or similarly less than 1 case per 1000 individuals diagnosed with GBD followed for 10 years. Of note, we identified a particularly high relative risk (5.76) of small bowel adenocarcinoma in individuals diagnosed with GBD between 20 and 39 years of age, suggesting that this group may benefit from increased small bowel cancer awareness in clinicians, but we found no trend for risk in other younger age groups, so this could potentially be a chance finding. However, given the relatively low absolute risk, our findings do not support a need for general surveillance of small bowel cancer in any subgroups of GBD patients.

### 4.1. Comparison to Previous Literature

One Swedish cohort study examined the excess small bowel cancer risk in cholecystectomy patients in year 1965–1997; the standardized incidence ratio (SIR) for adenocarcinoma was 1.77 95% CI = 1.37–2.24 and 1.71 95% CI = 1.39–2.08 for carcinoids [11]. A Danish cohort study (1977–1989) showed a relative small bowel cancer risk of 2.6 (95% CI = 1.6–3.9) in patients with gallstones, the risk being higher in women (2.84) compared to men (2.18) [10]. On the contrary, our study showed an increased risk of adenocarcinoma in men but not in women and consistently higher estimates for men than women for all outcomes. A more recent case-control study of 23 patients with small bowel adenocarcinoma from Switzerland presented an odds ratio (OR) of 3.96 95% CI = 1.10–14.3 [13] for cholelithiasis. The same study also pooled results from previous evidence (four studies whereof the two previously described were included) in a meta-analysis; the pooled relative risk of small intestinal cancer associated with cholelithiasis was 2.35 95% CI = 1.82–3.03 [13]. Another case-control study from the American SEER registries, including individuals diagnosed with small bowel cancer in 1992–2005, reported an OR of 1.27 95% CI = 1.01–1.60 for small intestinal carcinoids (79 cases) and OR 1.21 95% CI = 0.96–1.53 for adenocarcinomas (77 cases) [12] in patients with previous gallstones or cholecystectomy. In concordance with our results, the reported OR was higher the first 2 years from GBD diagnosis. Our study is thus the largest, based on 92 cases of adenocarcinomas and 81 cases of carcinoids in individuals with GBD, and also the first one to report risk estimates for small bowel adenomas. It is also the first cohort study that does not simply use SIR but uses matched comparators as comparison, which additionally allowed us to adjust for comorbidities and educational level. Further, it is the first study to explicitly report the absolute risk following gallbladder disease, adding large value in terms of clinical risk assessment.

### 4.2. Strengths and Limitations

The main strength of our study is the large number of GBD patients with virtually complete follow-up, for a median duration of 12 years. This allowed us to calculate precise risk for several clinically important outcomes. Overall, the positive predictive value (PPV) for most diagnoses in the Swedish Patient Register is about 85–95% [16]. The validity of GBD in our study is likely to be even higher, considering that we also requested a gallbladder biopsy for our GBD diagnosis. We are not aware of any validation of small bowel adenocarcinoma, but the Swedish Cancer Register has high sensitivity for “other digestive cancers” (96%) [23] and additionally our outcome was largely based on histopathology reports from ESPRESSO, allowing unprecedented specificity.

A limitation is that we did not have data on patient body mass index (BMI) or actual smoking pattern, but instead we used obesity data from the Patient Register, and a proxy for smoking/COPD to account for residual confounding. Neither did we have any data on GBD biomarkers.

## 5. Conclusions

In conclusion, we found that GBD increased the risk of small bowel adenocarcinomas, adenomas, and carcinoids. The association with carcinoids deserves to be studied in more detail and confirmed in larger material, since the induction time (11–16 years after GBD diagnosis) is consistent with carcinogenesis, suggesting a potential causal relationship.

## Figures and Tables

**Table 1:** Baseline characteristics of study cohort with gallbladder disease and small bowel adenocarcinoma, adenoma, and carcinoids (populations differ slightly due to exclusion of the outcome of interest prior to study entry).

Characteristic	Gallbladder Disease (*n* = 156,390)	Matched Comparators (*n* = 647,844)	Gallbladder Disease (*n* = 156,307)	Matched Comparators (*n* = 647,829)	Gallbladder Disease (*n* = 156,306)	Matched Comparators (*n* = 647,835)
Outcome	Adenocarcinoma		Adenoma		Carcinoid	
Women, no. (%)	97,859 (62.6%)	428,597 (63.5%)	97,826 (62.6%)	428,599 (63.5%)	97,821 (62.6%)	428,600 (63.5%)
Men, no. (%)	58,531 (37.4%)	246,236 (36.5%)	58,481 (37.4%)	246,230 (36.5%)	58,485 (37.4%)	246,235 (36.5%)
Age						
Mean (SD)	53.7 (14.9)	52.9 (15.1)	53.7 (14.9)	52.9 (15.1)	53.7 (14.9)	52.9 (15.1)
Median (IQR)	55.0 (42.0–66.0)	54.0 (41.0–65.0)	55.0 (42.0–66.0)	54.0 (41.0–65.0)	55.0 (42.0–66.0)	54.0 (41.0–65.0)
Range, min-max	20.0–79.0	20.0–79.0	20.0–79.0	20.0–79.0	20.0–79.0	20.0–79.0
Age (years), no. (%)						
20–39	31,963 (20.4%)	149,152 (22.1%)	31,958 (20.4%)	149,152 (22.1%)	31,961 (20.4%)	149,151 (22.1%)
40–49	28,076 (18.0%)	124,854 (18.5%)	28,069 (18.0%)	124,854 (18.5%)	28,066 (18.0%)	124,854 (18.5%)
50–59	34,384 (22.0%)	145,699 (21.6%)	34,368 (22.0%)	145,700 (21.6%)	34,369 (22.0%)	145,702 (21.6%)
60–69	35,445 (22.7%)	146,721 (21.7%)	35,417 (22.7%)	146,717 (21.7%)	35,410 (22.7%)	146,720 (21.7%)
70–79	26,522 (17.0%)	108,407 (16.1%)	26,495 (17.0%)	108,406 (16.1%)	26,500 (17.0%)	108,408 (16.1%)
Highest attained level of education (years), *n* (%)						
≤9	41,378 (26.5%)	153,416 (22.7%)	41,348 (26.5%)	153,412 (22.7%)	41,348 (26.5%)	153,418 (22.7%)
10–12	58,471 (37.4%)	241,837 (35.8%)	58,437 (37.4%)	241,836 (35.8%)	58,439 (37.4%)	241,836 (35.8%)
>12	30,320 (19.4%)	168,683 (25.0%)	30,308 (19.4%)	168,685 (25.0%)	30,305 (19.4%)	168,685 (25.0%)
Missing	26,221 (16.8%)	110,897 (16.4%)	26,214 (16.8%)	110,896 (16.4%)	26,214 (16.8%)	110,896 (16.4%)
Start year of follow-up						
1965–1989	20,309 (13.0%)	84,877 (12.6%)	20,306 (13.0%)	84,876 (12.6%)	20,305 (13.0%)	84,877 (12.6%)
1990–1999	46,787 (29.9%)	197,611 (29.3%)	46,769 (29.9%)	197,614 (29.3%)	46,762 (29.9%)	197,611 (29.3%)
2000–2009	56,001 (35.8%)	243,146 (36.0%)	55,966 (35.8%)	243,140 (36.0%)	55,955 (35.8%)	243,146 (36.0%)
2010–2017	33,293 (21.3%)	149,199 (22.1%)	33,266 (21.3%)	149,199 (22.1%)	33,284 (21.3%)	149,201 (22.1%)
Follow-up, years						
Mean (SD)	12.3 (8.1)	11.9 (8.1)	12.3 (8.1)	11.9 (8.1)	12.3 (8.1)	11.9 (8.1)
Median (IQR)	11.5 (5.6–17.8)	10.9 (5.2–17.4)	11.5 (5.6–17.8)	10.9 (5.2–17.4)	11.5 (5.6–17.8)	10.9 (5.2–17.4)
Range, min-max	0.0–46.0	0.0–46.0	0.0–46.0	0.0–46.0	0.0–46.0	0.0–46.0
Comorbidities (ever recorded in registries)						
Alcohol	5914 (3.8%)	1771 (0.3%)	5910 (3.8%)	1771 (0.3%)	5912 (3.8%)	1771 (0.3%)
COPD	13,367 (8.5%)	3558 (0.5%)	13,352 (8.5%)	3558 (0.5%)	13,359 (8.5%)	3558 (0.5%)
Ischemic heart disease	5255 (3.4%)	15,742 (2.3%)	5252 (3.4%)	15,741 (2.3%)	5252 (3.4%)	15,742 (2.3%)
Type 2 diabetes	7626 (4.9%)	17,145 (2.5%)	7612 (4.9%)	17,145 (2.5%)	7623 (4.9%)	17,146 (2.5%)
Obesity	4693 (3.0%)	4580 (0.7%)	4688 (3.0%)	4580 (0.7%)	4690 (3.0%)	4580 (0.7%)

COPD, chronic obstructive pulmonary disease.

**Table 2:** Stratified risk of small bowel adenocarcinoma in individuals with gallbladder disease and matched general population comparators.

Group	N (%)		N Events (%)		Incidence Rate (95% CI) Per 100,000 PY		HR * (95% CI)
	Gallbladder Disease	Comparators	Gallbladder Disease	Comparators	Gallbladder Disease	Comparators	
**Overall**	156,390 (100.0%)	674,833 (100.0%)	92 (0.1%)	260 (0.0%)	4.8 (3.8–5.8)	3.2 (2.8–3.6)	**1.42 (1.08–1.87)**
Follow-up (0- < 1 represents year 1- < 2 after GBD, as follow up starts at 1 year after GBD)							
0- < 1 y	156,381 (100.0%)	674,811 (100.0%)	15 (0.0%)	13 (0.0%)	9.8 (4.8–14.8)	2.0 (0.9–3.0)	**5.76 (2.31–14.40)**
1- < 5 y	149,668 (95.7%)	642,450 (95.2%)	18 (0.0%)	63 (0.0%)	3.3 (1.8–4.9)	2.7 (2.1–3.4)	1.16 (0.62–2.16)
5- < 10 y	121,425 (77.6%)	510,981 (75.7%)	23 (0.0%)	66 (0.0%)	4.4 (2.6–6.2)	3.0 (2.3–3.8)	1.48 (0.85–2.59)
10- < 15 y	88,010 (56.3%)	364,100 (54.0%)	19 (0.0%)	54 (0.0%)	5.3 (2.9–7.7)	3.7 (2.7–4.6)	1.28 (0.68–2.43)
15- < 20 y	54,823 (35.1%)	224,869 (33.3%)	9 (0.0%)	37 (0.0%)	4.4 (1.5–7.3)	4.4 (3.0–5.8)	0.98 (0.42–2.30)
≥20 y	28,662 (18.3%)	117,131 (17.4%)	8 (0.0%)	27 (0.0%)	5.4 (1.7–9.2)	4.5 (2.8–6.2)	1.39 (0.51–3.79)
Sex							
Women	97,859 (62.6%)	428,597 (63.5%)	47 (0.0%)	156 (0.0%)	3.7 (2.6–4.7)	2.9 (2.4–3.3)	1.20 (0.83–1.73)
Men	58,531 (37.4%)	246,236 (36.5%)	45 (0.1%)	104 (0.0%)	7.0 (4.9–9.0)	4.0 (3.2–4.7)	**1.80 (1.16–2.79)**
Age at gallbladder diagnosis							
20–39	31,963 (20.4%)	149,152 (22.1%)	7 (0.0%)	7 (0.0%)	1.5 (0.4–2.6)	0.3 (0.1–0.6)	**5.26 (1.57–17.63)**
40–49	28,076 (18.0%)	124,854 (18.5%)	6 (0.0%)	21 (0.0%)	1.6 (0.3–2.8)	1.3 (0.7–1.8)	0.99 (0.28–3.57)
50–59	34,384 (22.0%)	145,699 (21.6%)	24 (0.1%)	64 (0.0%)	5.3 (3.2–7.4)	3.5 (2.6–4.3)	1.51 (0.88–2.59)
60–69	35,445 (22.7%)	146,721 (21.7%)	34 (0.1%)	99 (0.1%)	8.5 (5.7–11.4)	6.2 (5.0–7.5)	1.38 (0.87–2.20)
70–79	26,522 (17.0%)	108,407 (16.1%)	21 (0.1%)	69 (0.1%)	9.0 (5.1–12.8)	7.6 (5.8–9.4)	1.19 (0.68–2.10)
Year of gallbladder diagnosis							
1965–1989	20,309 (13.0%)	84,877 (12.6%)	7 (0.0%)	17 (0.0%)	5.5 (3.3–7.7)	4.3 (3.3–5.3)	1.71 (0.58–5.06)
1990–1999	46,787 (29.9%)	197,611 (29.3%)	11 (0.0%)	24 (0.0%)	5.5 (3.9–7.1)	3.3 (2.7–4.0)	2.15 (0.95–4.87)
2000–2009	56,001 (35.8%)	243,146 (36.0%)	13 (0.0%)	28 (0.0%)	3.8 (2.2–5.3)	2.6 (2.0–3.2)	**2.36 (1.03–5.38)**
2010–2017	10,874 (7.0%)	48,476 (7.2%)	1 (0.0%)	5 (0.0%)	2.0 (0.0–4.9)	1.6 (0.4–2.8)	0.72 (0.05–11.20)
Year–follow-up restricted to first 5 years of follow-up							
1965–1989	4255 (8.8%)	21,396 (8.9%)	1 (0.0%)	1 (0.0%)	7.2 (1.9–12.5)	4.2 (2.2–6.2)	3.87 (0.24–63.34)
1990–1999	13,291 (27.6%)	66,455 (27.8%)	6 (0.0%)	4 (0.0%)	4.8 (2.0–7.7)	2.5 (1.5–3.5)	NE
2000–2009	19,601 (40.7%)	96,967 (40.5%)	4 (0.0%)	9 (0.0%)	4.7 (2.2–7.3)	2.4 (1.5–3.2)	2.31 (0.65–8.24)
2010	4083 (8.5%)	20,318 (8.5%)	1 (0.0%)	1 (0.0%)	2.0 (0.0–5.8)	2.2 (0.3–4.2)	2.45 (0.15–39.72)
Level of education							
≤9 years	41,378 (26.5%)	153,416 (22.7%)	32 (0.1%)	80 (0.1%)	6.9 (4.5–9.3)	4.8 (3.7–5.8)	1.62 (0.88–2.97)
10–12 years	58,471 (37.4%)	241,837 (35.8%)	20 (0.0%)	59 (0.0%)	3.2 (1.8–4.6)	2.3 (1.7–2.9)	0.92 (0.40–2.10)
>12 years	30,320 (19.4%)	168,683 (25.0%)	14 (0.0%)	34 (0.0%)	4.6 (2.2–7.0)	2.0 (1.3–2.7)	1.47 (0.47–4.54)
Education missing	26,221 (16.8%)	110,897 (16.4%)	26 (0.1%)	87 (0.1%)	4.9 (3.0–6.7)	4.0 (3.2–4.9)	1.25 (0.75–2.07)

* Conditioned on matching set (age, sex, county, and calendar period) and further adjusted for highest attained education, ischemic heart disease, obesity, type 2 diabetes, a proxy for smoking, and alcohol use. Bold HR marks statistically significant estimate. NE—not estimated due to lack of relevant strata, i.e., case censored in strata with matched comparator events.

**Table 3:** Stratified risk of small bowel adenomas in individuals with gallbladder disease and matched general population comparators.

Group	N (%)		N Events (%)		Incidence Rate (95% CI) Per 100,000 PY		HR * (95% CI)
	Gallbladder Disease	Comparators	Gallbladder Disease	Comparators	Gallbladder Disease	Comparators	
**Overall**	156,307 (100.0%)	674,829 (100.0%)	132 (0.1%)	260 (0.0%)	6.9 (5.7–8.0)	3.2 (2.8–3.6)	**1.79 (1.41–2.27)**
Follow-up							
0- < 1 y	156,298 (100.0%)	674,807 (100.0%)	9 (0.0%)	15 (0.0%)	5.9 (2.0–9.7)	2.3 (1.1–3.4)	2.49 (0.99–6.27)
1- < 5 y	149,590 (95.7%)	642,434 (95.2%)	37 (0.0%)	51 (0.0%)	6.8 (4.6–9.0)	2.2 (1.6–2.8)	**2.69 (1.68–4.31)**
5- < 10 y	121,351 (77.6%)	510,951 (75.7%)	34 (0.0%)	71 (0.0%)	6.5 (4.3–8.7)	3.3 (2.5–4.0)	1.60 (0.99–2.60)
10- < 15 y	87,964 (56.3%)	364,055 (53.9%)	25 (0.0%)	63 (0.0%)	7.0 (4.3–9.7)	4.3 (3.2–5.3)	1.51 (0.88–2.62)
15- < 20 y	54,800 (35.1%)	224,826 (33.3%)	12 (0.0%)	36 (0.0%)	5.9 (2.5–9.2)	4.3 (2.9–5.7)	0.99 (0.43–2.32)
≥20 y	28,650 (18.3%)	117,111 (17.4%)	15 (0.1%)	24 (0.0%)	10.2 (5.0–15.3)	4.0 (2.4–5.6)	1.70 (0.77–3.74)
Sex							
Women	97,826 (62.6%)	428,599 (63.5%)	69 (0.1%)	148 (0.0%)	5.4 (4.1–6.7)	2.7 (2.3–3.2)	**1.66 (1.19–2.30)**
Men	58,481 (37.4%)	246,230 (36.5%)	63 (0.1%)	112 (0.0%)	9.8 (7.3–12.2)	4.3 (3.5–5.0)	**1.91 (1.34–2.71)**
Age at gallbladder diagnosis							
20–39	31,958 (20.4%)	149,152 (22.1%)	6 (0.0%)	15 (0.0%)	1.3 (0.3–2.3)	0.7 (0.4–1.1)	1.68 (0.56–4.98)
40–49	28,069 (18.0%)	124,854 (18.5%)	23 (0.1%)	37 (0.0%)	6.0 (3.6–8.5)	2.3 (1.5–3.0)	**2.04 (1.13–3.69)**
50–59	34,368 (22.0%)	145,700 (21.6%)	36 (0.1%)	66 (0.0%)	8.0 (5.4–10.6)	3.6 (2.7–4.5)	**1.64 (1.01–2.66)**
60–69	35,417 (22.7%)	146,717 (21.7%)	38 (0.1%)	87 (0.1%)	9.5 (6.5–12.6)	5.5 (4.3–6.6)	**1.58 (1.01–2.49)**
70–79	26,495 (17.0%)	108,406 (16.1%)	29 (0.1%)	55 (0.1%)	12.4 (7.9–17.0)	6.0 (4.4–7.6)	**2.08 (1.23–3.49)**
Year of gallbladder diagnosis							
1965–1989	20,306 (13.0%)	84,876 (12.6%)	22 (0.1%)	57 (0.1%)	5.0 (2.9–7.1)	3.2 (2.4–4.1)	1.07 (0.59–1.96)
1990–1999	46,769 (29.9%)	197,614 (29.3%)	47 (0.1%)	112 (0.1%)	5.8 (4.2–7.5)	3.3 (2.7–4.0)	**1.53 (1.03–2.27)**
2000–2009	55,966 (35.8%)	243,140 (36.0%)	58 (0.1%)	83 (0.0%)	9.9 (7.4–12.4)	3.3 (2.6–4.0)	**2.73 (1.86–3.99)**
2010–2017	33,266 (21.3%)	149,199 (22.1%)	5 (0.0%)	8 (0.0%)	5.1 (0.6–9.6)	1.8 (0.6–3.1)	2.47 (0.62–9.80)
Year–follow-up restricted to first 5 years of follow-up							
1965–1989	20,306 (13.0%)	84,876 (12.6%)	3 (0.0%)	4 (0.0%)	3.1 (0.0–6.5)	1.0 (0.0–2.0)	2.27 (0.37–13.90)
1990–1999	46,769 (29.9%)	197,614 (29.3%)	14 (0.0%)	23 (0.0%)	6.2 (2.9–9.4)	2.4 (1.4–3.4)	2.00 (0.95–4.21)
2000–2009	55,966 (35.8%)	243,140 (36.0%)	24 (0.0%)	31 (0.0%)	8.8 (5.3–12.3)	2.6 (1.7–3.5)	**3.32 (1.85–5.96)**
2010	10,865 (7.0%)	48,479 (7.2%)	3 (0.0%)	2 (0.0%)	5.9 (0.0–12.6)	0.9 (0.0–2.1)	2.41 (0.16–35.48)
Level of education							
≤9 years	41,348 (26.5%)	153,412 (22.7%)	54 (0.1%)	68 (0.0%)	11.6 (8.5–14.7)	4.1 (3.1–5.0)	**3.82 (2.03–7.19)**
10–12 years	58,437 (37.4%)	241,836 (35.8%)	37 (0.1%)	63 (0.0%)	5.9 (4.0–7.8)	2.5 (1.9–3.1)	**2.23 (1.13–4.40)**
>12 years	30,308 (19.4%)	168,685 (25.0%)	16 (0.1%)	58 (0.0%)	5.3 (2.7–7.9)	3.5 (2.6–4.3)	2.25 (0.83–6.10)
Education missing	26,214 (16.8%)	110,896 (16.4%)	25 (0.1%)	71 (0.1%)	4.7 (2.8–6.5)	3.3 (2.5–4.1)	0.93 (0.53–1.65)

* Conditioned on matching set (age, sex, county, and calendar period) and further adjusted for highest attained education, ischemic heart disease, obesity, type 2 diabetes, a proxy for smoking, and alcohol use. Bold HR marks statistically significant estimate.

**Table 4:** Stratified risk of carcinoids in individuals with gallbladder disease and matched general population comparators.

Group	N (%)		N Events (%)		Incidence Rate (95% CI) Per 100,000 PY		HR * (95% CI)
	Gallbladder Disease	Comparators	Gallbladder Disease	Comparators	Gallbladder Disease	Comparators	
**Overall**	156,306 (100.0%)	674,835 (100.0%)	81 (0.1%)	148 (0.0%)	4.2 (3.3–5.1)	1.8 (1.5–2.1)	**2.07 (1.52–2.81)**
Follow-up							
0- < 1 y	156,296 (100.0%)	674,813 (100.0%)	2 (0.0%)	12 (0.0%)	1.3 (0.0–3.1)	1.8 (0.8–2.9)	0.48 (0.06–3.86)
1- < 5 y	149,593 (95.7%)	642,443 (95.2%)	24 (0.0%)	35 (0.0%)	4.4 (2.7–6.2)	1.5 (1.0–2.0)	**3.01 (1.70–5.34)**
5- < 10 y	121,360 (77.6%)	510,970 (75.7%)	24 (0.0%)	38 (0.0%)	4.6 (2.8–6.4)	1.7 (1.2–2.3)	1.81 (0.98–3.31)
10- < 15 y	87,970 (56.3%)	364,079 (54.0%)	18 (0.0%)	26 (0.0%)	5.0 (2.7–7.4)	1.8 (1.1–2.4)	**3.28 (1.58–6.78)**
15- < 20 y	54,801 (35.1%)	224,857 (33.3%)	8 (0.0%)	18 (0.0%)	3.9 (1.2–6.6)	2.1 (1.2–3.1)	1.56 (0.59–4.13)
≥20 y	28,650 (18.3%)	117,133 (17.4%)	4 (0.0%)	19 (0.0%)	2.7 (0.1–5.4)	3.2 (1.7–4.6)	1.01 (0.27–3.69)
Sex							
Women	97,821 (62.6%)	428,600 (63.5%)	46 (0.0%)	82 (0.0%)	3.6 (2.6–4.6)	1.5 (1.2–1.8)	**1.95 (1.29–2.94)**
Men	58,485 (37.4%)	246,235 (36.5%)	35 (0.1%)	66 (0.0%)	5.4 (3.6–7.2)	2.5 (1.9–3.1)	**2.07 (1.29–3.33)**
Age at gallbladder diagnosis							
20–39	31,961 (20.4%)	149,151 (22.1%)	6 (0.0%)	5 (0.0%)	1.3 (0.3–2.3)	0.2 (0.0–0.4)	3.45 (0.80–14.95)
40–49	28,066 (18.0%)	124,854 (18.5%)	12 (0.0%)	19 (0.0%)	3.1 (1.4–4.9)	1.2 (0.6–1.7)	**2.98 (1.32–6.74)**
50–59	34,369 (22.0%)	145,702 (21.6%)	24 (0.1%)	43 (0.0%)	5.3 (3.2–7.4)	2.3 (1.6–3.0)	**1.93 (1.09–3.42)**
60–69	35,410 (22.7%)	146,720 (21.7%)	27 (0.1%)	56 (0.0%)	6.8 (4.2–9.3)	3.5 (2.6–4.4)	**2.08 (1.26–3.44)**
70–79	26,500 (17.0%)	108,408 (16.1%)	12 (0.0%)	25 (0.0%)	5.1 (2.2–8.1)	2.7 (1.7–3.8)	1.14 (0.45–2.90)
Year of gallbladder diagnosis							
1965–1989	20,305 (13.0%)	84,877 (12.6%)	14 (0.1%)	36 (0.0%)	3.2 (1.5–4.9)	2.0 (1.4–2.7)	1.62 (0.80–3.30)
1990–1999	46,762 (29.9%)	197,611 (29.3%)	38 (0.1%)	66 (0.0%)	4.7 (3.2–6.2)	2.0 (1.5–2.4)	**1.97 (1.25–3.11)**
2000–2009	55,955 (35.8%)	243,146 (36.0%)	25 (0.0%)	41 (0.0%)	4.3 (2.6–5.9)	1.6 (1.1–2.1)	**2.17 (1.23–3.83)**
2010–2017	33,284 (21.3%)	149,201 (22.1%)	4 (0.0%)	5 (0.0%)	4.1 (0.1–8.1)	1.1 (0.1–2.2)	**4.72 (1.06–21.04)**
Year–follow-up restricted to first 5 years of follow-up							
1965–1989	20,305 (13.0%)	84,877 (12.6%)	1 (0.0%)	7 (0.0%)	1.0 (0.0–3.0)	1.7 (0.5–3.0)	1.07 (0.12–9.62)
1990–1999	46,762 (29.9%)	197,611 (29.3%)	10 (0.0%)	17 (0.0%)	4.4 (1.7–7.1)	1.8 (0.9–2.6)	**2.58 (1.06–6.26)**
2000–2009	55,955 (35.8%)	243,146 (36.0%)	12 (0.0%)	18 (0.0%)	4.4 (1.9–6.9)	1.5 (0.8–2.2)	**2.47 (1.06–5.72)**
2010	10,873 (7.0%)	48,479 (7.2%)	1 (0.0%)	2 (0.0%)	2.0 (0.0–5.8)	0.9 (0.0–2.1)	3.32 (0.15–74.41)
Level of education							
≤9 years	41,348 (26.5%)	153,418 (22.7%)	20 (0.0%)	48 (0.0%)	4.3 (2.4–6.2)	2.9 (2.1–3.7)	1.88 (0.85–4.15)
10–12 years	58,439 (37.4%)	241,836 (35.8%)	31 (0.1%)	37 (0.0%)	5.0 (3.2–6.7)	1.5 (1.0–1.9)	**4.89 (2.14–11.16)**
>12 years	30,305 (19.4%)	168,685 (25.0%)	13 (0.0%)	20 (0.0%)	4.3 (2.0–6.6)	1.2 (0.7–1.7)	0.70 (0.13–3.70)
Education missing	26,214 (16.8%)	110,896 (16.4%)	17 (0.1%)	43 (0.0%)	3.2 (1.7–4.7)	2.0 (1.4–2.6)	1.62 (0.84–3.12)

* Conditioned on matching set (age, sex, county, and calendar period) and further adjusted for highest attained education, ischemic heart disease, obesity, type 2 diabetes, a proxy for smoking, and alcohol use. Bold HR marks statistically significant estimate.

## Data Availability

Data availability requests can be sent to the corresponding author.

## Abbreviations

BMI	body mass index
CI	confidence interval
COPD	chronic obstructive pulmonary disease
ESPRESSO	epidemiology strengthened by histopathology reports in Sweden
GBD	gallbladder disease
GI	gastrointestinal
HR	hazard ratio
OR	odds ratio
SnoMed	systematized nomenclature of medicine

## Appendix A. CD-SBA

Codes used to identify gallbladder disease (GBD) from the Swedish patient registry: ICD10: K80, K81, ICD-9:574, 575 plus procedure codes for cholecystectomy: ICD10: JKA20, JKA21 and ICD-9: 535.

The following ICD-8/9/10 codes were used to extract comorbidities from the Swedish patient registry: type 2 diabetes (ICD10: E11-14, ICD8&9: 250); obesity (ICD10: E66: ICD 8 &9: 278A, 278B); ischemic heart disease (ICD10: I21, I22, I25.2, ICD8&9: 410, 411, 412); alcohol (ICD10: E24.4: F10, G62.1, I42.6, K29.2, G31.2, G71.2, K70, K85.2, K86.0, =35.4, ICD9: 291, 303, 357F, 425F, 535D, 571(A,B,C,D), ICD8: 261,262, 291, 303, 571.0, 571.1); proxy for heavy smoking incl. chronic obstructive pulmonary disease (ICD10: J41-J44, Z72, Z71.6, F17, ICD9: 491, 492, 496, 305.1, V15.8, ICD8: 490, 491, 492, 495).

**Table A1:** Definition of exposure and outcomes from ESPRESSO.

Characteristics	SnoMed Code	Topography
Gallbladder disease	M0X, M3X, M4X, M5X (all non-malignant gallbladder-related conditions–X represents any numbers)	T57
Adenoma	M82632, M82112, M82611, M81400, M81400, M72040, M82612, M82630, M82100, M82102	T64 and T65
Adenocarcinoma	M81403	T64 and T65
Carcinoid	M82403, M82463, M82493	T64 and T65
